# Aerobic exercise, sleep quality and anxiety among perimenopausal women

**DOI:** 10.6026/973206300221496

**Published:** 2026-03-31

**Authors:** Shweta Gupta, Alok Ranjan, Poonam Motwani

**Affiliations:** 1 Department of Physiology, Prasad Institute of Medical Sciences, Lucknow, Uttar Pradesh, India; 2 Department of Ophthalmology, Dr. Ram Manohar Lohia Institute of Medical Sciences (Dr. RMLIMS), Lucknow, Uttar Pradesh, India; 3 Department of Physiology, Government Medical College (GMC), Ratlam, Madhya Pradesh, India

**Keywords:** Anxiety, aerobic exercise, perimenopause, sleep quality, women

## Abstract

Perimenopause is a critical phase in a woman's life, marked by hormonal fluctuations that often lead to sleep disturbances and increased anxiety. These symptoms can significantly affect a woman's quality of life, making effective interventions necessary. Therefore, it is of interest to assess the impact of aerobic exercise on sleep quality and anxiety levels in perimenopausal women. After a 12-week intervention, results indicated significant improvements in both sleep quality and anxiety in the experimental group. The study advances understanding of non-pharmacological interventions, suggesting aerobic exercise as a valuable tool for managing perimenopausal symptoms.

## Background:

Perimenopause is a natural stage in a woman's life, typically occurring between the ages of 40 and 55, marking the transition from the reproductive phase to menopause. This period is characterized by hormonal fluctuations, particularly a decline in estrogen levels, which can lead to a range of physical, emotional and psychological symptoms [1]. Among the most prevalent issues during perimenopause are sleep disturbances and increased anxiety, which significantly impact a woman's overall well-being. Studies have shown that up to 60% of women experience sleep problems during this time, with insomnia being the most common complaint [2]. Similarly, anxiety levels tend to increase, with some women reporting feelings of nervousness, irritability and worry. These issues can affect daily functioning and quality of life, making it crucial to find effective interventions to manage these symptoms [3]. Sleep quality refers to how well an individual sleep, encompassing factors such as duration, restfulness and the absence of disturbances during sleep [4]. Aerobic exercise, defined as any physical activity that increases heart rate and promotes cardiovascular endurance, has long been recognized for its numerous health benefits. These include improvements in cardiovascular health, muscle strength, flexibility and endurance. In addition, emerging research suggests that aerobic exercise may play a significant role in alleviating common perimenopausal symptoms, including poor sleep quality and anxiety. Regular aerobic activities such as walking, jogging, swimming and cycling have been found to promote better sleep by helping to regulate circadian rhythms, reduce stress levels and promote relaxation [5]. Exercise is known to trigger the release of endorphins; neurotransmitters that help alleviate pain and promote a sense of well-being, which may further contribute to improved sleep and reduced anxiety [6].

The relationship between aerobic exercise and sleep quality has been extensively studied, with results indicating that moderate- intensity aerobic activity can significantly improve sleep in various populations, including older adults and those with chronic sleep disorders [7]. Moreover, exercise-induced changes in brain chemistry, such as increased serotonin levels, are believed to contribute to both improved mood and sleep. When it comes to anxiety, several studies have shown that aerobic exercise can reduce symptoms by reducing the body's physiological response to stress and by enhancing mental clarity. Furthermore, regular exercise has been linked to increased cognitive function, emotional regulation and better stress management, all of which are critical in managing anxiety levels [8]. For women in the perimenopausal phase, the hormonal changes that accompany this transition often exacerbate anxiety and sleep disturbances. This can create a cyclical pattern where poor sleep and heightened anxiety feed into one another, worsening the overall quality of life [9]. As a non- invasive and cost-effective intervention, aerobic exercise presents a viable alternative to pharmacological treatments, which may have undesirable side effects. In recent years, there has been growing interest in understanding the potential of exercise interventions to manage perimenopausal symptoms, with several studies showing promising results [10]. Therefore, it is of interest to determine the role of aerobic exercise in improving sleep quality and anxiety in perimenopausal women.

## Methodology:

This study aimed to investigate the role of aerobic exercise in improving sleep quality and anxiety levels among perimenopausal women. The research design adopted was a quasi-experimental, pre-test and post-test intervention study, with a control group. This design allowed for an assessment of the effects of aerobic exercise on the variables of interest (sleep quality and anxiety) within a specified period.

## Participants:

The study recruited 100 perimenopausal women aged 40 to 55 years, who were experiencing sleep disturbances and/or anxiety symptoms. Participants were selected using convenience sampling from local healthcare clinics and community centers.

## The Inclusion criteria for participants were:

[1] Women aged 40 to 55 years, in the perimenopausal stage, confirmed by clinical assessment.

[2] Self-reported issues with sleep quality (e.g., insomnia, difficulty falling asleep or waking up frequently).

[3] Self-reported anxiety symptoms as indicated by scores on an anxiety scale (such as the Generalized Anxiety Disorder-7, GAD-7).

[4] No major medical conditions or contraindications for aerobic exercise (e.g., cardiovascular disease, recent surgery).

[5] Not currently engaging in regular aerobic exercise (defined as exercising for less than 30 minutes, three times per week).

## Exclusion criteria included:

[1] Women with severe psychiatric disorders (e.g., major depression or psychosis).

[2] Women undergoing hormone replacement therapy or taking medications that could significantly affect sleep or anxiety levels.

## Research design:

This study employed a pre-test and post-test intervention design with a control group. Participants were randomly assigned to one of two groups: an experimental group that engaged in a structured aerobic exercise program and a control group that did not receive any intervention.

## Pre-Test:

Before the intervention, participants completed baseline assessments for sleep quality and anxiety levels.

The following tools were used:

[1] Sleep quality assessment: The Pittsburgh Sleep Quality Index (PSQI) was used to assess participants' sleep quality. This questionnaire included 19 items that assessed sleep duration, sleep disturbances, sleep latency and sleep efficiency. A global score of 5 or more indicated poor sleep quality.

[2] Anxiety assessment: The Generalized Anxiety Disorder-7 (GAD-7) was used to assess anxiety symptoms. This 7-item scale measured the severity of generalized anxiety, with scores ranging from 0 to 21. A score of 10 or higher indicated moderate to severe anxiety.

## Intervention:

The experimental group engaged in a 12-week aerobic exercise program, consisting of moderate-intensity exercise three times per week.

Each session lasted 45 minutes and included

[1] Warm-up: 5 minutes of light walking and stretching.

[2] Aerobic Exercise: 35 minutes of moderate-intensity aerobic activities such as walking, cycling, or low-impact aerobics. Intensity was monitored using the Borg Rating of Perceived Exertion (RPE) scale, ensuring participants stayed at a moderate intensity level (RPE 12-14).

[3] Cool-down: 5 minutes of stretching and relaxation exercises.

Participants in the control group did not engage in any structured exercise program but were asked to maintain their usual routine during the study period.

## Post-test:

At the end of the 12-week period, both groups completed the same assessments as in the pre-test to measure changes in sleep quality and anxiety levels. The PSQI and GAD-7 were administered again to evaluate the outcomes of the intervention.

## Data collection:

Data were collected at two time points: at baseline (pre-test) and after the intervention (post-test). In addition to the main outcome measures (PSQI and GAD-7), demographic data such as age, education and medical history were collected to describe the study sample and control for potential confounders.

## Data analysis:

Data were analyzed using statistical software (SPSS or R). Descriptive statistics (means, standard deviations) were calculated for all variables. To assess the impact of the aerobic exercise intervention on sleep quality and anxiety, paired sample t-tests were used to compare pre-test and post-test scores within each group. An independent t-test was used to compare the changes between the experimental and control groups. The significance level was set at p < 0.05.

## Ethical considerations:

This study adhered to ethical standards as outlined by the Declaration of Helsinki. Informed consent was obtained from all participants before participation. Participants were informed of their right to withdraw from the study at any time without any consequences. Confidentiality was maintained by anonymizing participant data and all records were securely stored.

## Results:

The results of this study aimed to evaluate the impact of aerobic exercise on sleep quality and anxiety levels among perimenopausal women. After a 12-week intervention period, data were collected to compare the pre- and post-test scores for both sleep quality and anxiety levels. The experimental group engaged in the aerobic exercise program, while the control group maintained their usual routine. The data were analyzed using paired sample t-tests and independent t-tests to assess the changes within and between groups. The results showed a significant improvement in sleep quality among the experimental group, as indicated by a decrease in the Pittsburgh Sleep Quality Index (PSQI) scores. The mean PSQI score for the experimental group decreased from 9.7 (pre-test) to 6.1 (post-test), suggesting improved sleep quality. In contrast, the control group showed a minimal change, with mean PSQI scores remaining at 9.5 pre-test and 9.4 post-test (Table 1 - see PDF). A paired sample t-test revealed that the reduction in PSQI scores for the experimental group was statistically significant (p = 0.001), indicating that the aerobic exercise intervention significantly improved sleep quality. The control group did not show any significant change in their PSQI scores (p = 0.82), suggesting no improvement in sleep quality. Anxiety levels, as measured by the Generalized Anxiety Disorder-7 (GAD-7) scale, also showed a significant reduction in the experimental group. The mean GAD-7 score for the experimental group decreased from 14.2 (pre-test) to 8.4 (post-test), indicating a reduction in anxiety levels. In contrast, the control group showed minimal change, with mean GAD-7 scores remaining at 13.8 pre-test and 13.7 post-test (Table 2 - see PDF). A paired sample t-test revealed that the reduction in GAD-7 scores for the experimental group was statistically significant (p = 0.002), indicating that aerobic exercise effectively reduced anxiety. The control group did not show any significant change in their GAD-7 scores (p = 0.78), suggesting no reduction in anxiety levels. An independent t-test was conducted to compare the changes in sleep quality and anxiety between the experimental and control groups. The results indicated that the experimental group showed a significantly greater improvement in both sleep quality and anxiety compared to the control group. The independent t-test revealed that the experimental group experienced a significantly greater reduction in PSQI scores compared to the control group (p = 0.001), further supporting the effectiveness of aerobic exercise in improving sleep quality (Table 3 - see PDF).

Similarly, the experimental group showed a significantly greater reduction in GAD-7 scores compared to the control group (p = 0.002), indicating that aerobic exercise was more effective in reducing anxiety levels than no intervention (Table 4 - see PDF). The experimental group demonstrated significant improvements in both sleep quality and anxiety levels following the 12-week aerobic exercise intervention. In contrast, the control group did not show significant changes in either of the outcomes. The results of the study suggest that aerobic exercise can serve as an effective, non-pharmacological intervention to manage sleep disturbances and anxiety during perimenopause. These findings support the growing body of evidence on the benefits of exercise for improving mental and physical health, particularly during the perimenopausal transition.

## Discussion:

The results of this study demonstrated that 12 weeks of structured aerobic exercise significantly improved sleep quality and reduced anxiety levels among perimenopausal women. These findings are in alignment with several prior research studies, reinforcing the beneficial role of physical activity specifically aerobic exercise as a non pharmacological intervention for addressing common perimenopausal symptoms. A study by Zhao *et al.* (2022) [11] found that aerobics training significantly improved sleep quality, reduced anxiety and alleviated depressive symptoms in perimenopausal women after an 8 week intervention, supporting the current findings that regular aerobic activity can positively influence both sleep and mental health indicators in this population. Similarly, a systematic meta analysis by Zhao *et al.* (2023) [12] reported that exercise interventions including aerobic exercise significantly improved sleep quality and reduced insomnia symptoms among perimenopausal women. Although the meta analysis incorporated various forms of exercise (such as Qigong and yoga), the overall evidence suggested a positive effect of physical activity on sleep, which aligns with the improvements we observed in PSQI scores. Additional evidence comes from Mansikkamäki *et al.* (2023) [13] who reported that aerobic training over six months improved sleep quality among symptomatic menopausal women, suggesting that longer durations of cardiovascular exercise may be particularly effective in ameliorating sleep disturbances associated with hormonal changes. Research on the broader menopausal population further supports our findings. In a systematic evaluation, aerobic exercise was shown to effectively improve sleep disorders in menopausal women, with interventions demonstrating significant reductions in sleep disturbances compared with control conditions. Although this study pooled many menopausal populations rather than focusing solely on perimenopause, it underscores the potential of aerobic exercise to positively impact sleep health across climacteric phases [14]. While most studies corroborate the positive impact of exercise on sleep and psychological well being, some research has reported mixed outcomes depending on intervention specifics. For example, Sternfeld *et al.* (2014) [15] found that although moderate intensity aerobic training improved certain aspects of sleep and mood in menopausal women, the effects on vasomotor symptoms were less pronounced and sometimes did not reach statistical significance after adjustments. This variability highlights that while aerobic exercise is generally beneficial, factors such as adherence, exercise intensity and duration may influence the magnitude of effects. Several physiological and psychological mechanisms may explain the beneficial effects observed in this study. Aerobic exercise has been shown to enhance thermoregulation, reduce sympathetic nervous system activation and promote neurochemical changes (such as increased serotonin and endorphin release), all of which can contribute to improved sleep regulation and reduced anxiety. Additionally, the mood enhancing effects of exercise which include decreased stress hormone levels and improved emotional regulation likely contributed to the reductions in anxiety scores observed in the experimental group [16].

## Limitations:

One potential limitation of this study was the reliance on self-reported data for sleep quality and anxiety, which may have been subject to bias. Additionally, the study's findings may not be generalizable to all perimenopausal women, as the sample was restricted to those living in a specific geographic area. Finally, as a quasi-experimental design, the study may not have fully accounted for all confounding variables that could influence sleep and anxiety outcomes.

## Conclusion:

We show that aerobic exercise significantly improved sleep quality and reduced anxiety in perimenopausal women. The findings support the use of aerobic exercise as a non-pharmacological intervention for managing perimenopausal symptoms. Therefore, incorporating regular aerobic activity may serve as a beneficial strategy for improving overall well-being during perimenopause.
